# Evaluation of biochemical and hematological parameters in adults with Down syndrome

**DOI:** 10.1038/s41598-020-70719-2

**Published:** 2020-08-13

**Authors:** David de Gonzalo-Calvo, Isabel Barroeta, Madalina Nicoleta Nan, José Rives, Diana Garzón, María Carmona-Iragui, Bessy Benejam, Laura Videla, Susana Fernández, Miren Altuna, Sílvia Valldeneu, Rafael Blesa, Alberto Lleó, Francisco Blanco-Vaca, Juan Fortea, Mireia Tondo

**Affiliations:** 1 Biomedical Research Institute Sant Pau (IIB Sant Pau), Barcelona, Spain; 2 Institute of Biomedical Research of Barcelona (IIBB), Spanish National Research Council (CSIC), Barcelona, Spain; 3 Translational Research in Respiratory Medicine, University Hospital Arnau de Vilanova and Santa Maria, IRBLleida, Lleida, Spain; 4 Sant Pau Memory Unit, Department of Neurology, Hospital de La Santa Creu i Sant Pau, Biomedical Research Institute (IIB) Sant Pau, Universitat Autònoma de Barcelona, Barcelona, Spain; 5 Center of Biomedical Investigation Network for Neurodegenerative Diseases (CIBERNED), Madrid, Spain; 6 Department of Biochemistry, Hospital de La Santa Creu i Sant Pau, Biomedical Research Institute (IIB) Sant Pau, C/Sant Quintí 89, 08041 Barcelona, Spain; 7 Barcelona Down Medical Center, Fundació Catalana de Síndrome de Down, Barcelona, Spain; 8 Center of Biomedical Investigation Network for Diabetes and Metabolic Diseases (CIBERDEM), Madrid, Spain; 9 Department of Biochemistry and Molecular Biology, Universitat Autònoma de Barcelona, Barcelona, Spain

**Keywords:** Biomarkers, Diseases, Health care, Neurology

## Abstract

Down syndrome (DS) is the most common worldwide cause of intellectual disability of genetic origin and the most common chromosomal disorder affecting live-born infants. In addition to intellectual disability, individuals with DS have other comorbidities and complex medical conditions. The increase in the life expectancy of patients with DS requires expanding the knowledge about their clinical characteristics and related laboratory parameters. Several studies exploring laboratory tests in DS patients exist, but their focus is limited to specific areas of metabolism. Therefore, our main goal was to describe the biochemical and hematological findings in a DS cohort and to compare the values to those of a control population. A total of 248 DS individuals and 84 control subjects were enrolled. DS individuals had a higher frequency of several clinical conditions compared to control individuals and presented with significant differences with respect to the controls in both biochemical and hematological parameters. We found age- and sex-related differences in several of the parameters. A good understanding of the differences in our cohort might be of aid in the clinical follow-up of adults with DS, especially considering that the lifespan of DS individuals may reach 60 years of age in developed countries.

## Introduction

Down syndrome (DS) is the most common worldwide cause of intellectual disability of genetic origin and the most common chromosomal disorder affecting live-born infants, with an estimated birth prevalence of 14 per 10,000 live births^1–3^. Despite the shorter life expectancy when compared to healthy subjects and adults with other causes of intellectual disability^4^, there has been a progressive increase in the life expectancy of patients with DS in recent decades, currently reaching nearly 60 years^5^. This fact has increased the need to expand the knowledge about the clinical characteristics of DS individuals and the health problems differentiating them from both pediatric and adult populations^6^. DS is associated with a distinct phenotype involving many body systems. In addition to intellectual disability, individuals with DS present with a high number of comorbidities and complex medical conditions whose frequencies are modified throughout the lifespan of the individuals^7^. The increase in life expectancy has led to a higher prevalence of age-related pathologies, including premature Alzheimer’s disease (AD)^8^.

Since optimal medical management is associated with improved quality of life and functioning among persons with DS^9,10^, medical professionals, including pediatricians and other physicians, should closely supervise this population throughout their lifespan and evaluate their laboratory results. Previous investigations in DS cohorts have focused on select biochemical parameters, such as uric acid and thyroid function biomarkers, bone mineral density, nutritional zinc status, gonadal and endocrine function and glucose and lipid metabolism parameters^11–16^. However, no previous work has described a comprehensive panel of biochemical and hematological parameters in a large cohort of DS patients.

Our hypothesis is that a thorough analysis of the biochemical and hematological parameters will provide a basis to establish whether commonly observed alterations in DS individuals are intrinsic of the disease or have clinical implications similarly as for the general population. Therefore, our goals were to describe the biochemical and hematological findings in our DS cohort and to compare the values to those of a control population.

## Material and methods

### Study participants

This was a single-center descriptive study of adults with DS recruited at Barcelona Down Medical Center (Fundació Catalana Síndrome de Down and Hospital de la Santa Creu i Sant Pau, Barcelona) in Catalonia, Spain, according to a population-based health plan to screen for neurological comorbidities^17,18^. The Down Medical Center provides medical care specifically for individuals with DS and possesses over 2,500 medical records (more than 50% of the estimated Down syndrome population in Catalonia); therefore, it reflects the population with DS in our geographic area. The period of patient recruitment for this study was February 1, 2013, to June 30, 2018. In adults with DS (≥ 18 years), a biochemical and hematological analysis was performed as part of their annual health plan visit. A total of 254 patients were enrolled in the study. Six further patients were ultimately excluded for presenting with conditions unrelated to DS according to their medical records: 4 patients with hepatitis C, 1 patient with hepatitis B and 1 patient with breast cancer, resulting in a final total number of 248 DS individuals included (age range 18–63 years). A total of 84 healthy control participants in the same age range (23–65 years) were enrolled in the study. Volunteers were recruited from the SPIN (Sant Pau Initiative on Neurodegeneration) cohort (https://santpaumemoryunit.com/our-research/spin-cohort/) or social media (@SantPauMemory). Further details on the clinical protocol of the SPIN cohort can be found elsewhere^19^.

Based on current guidelines^17,20^, associated clinical conditions were obtained through a systematic review of the medical records, including the following: history of arterial hypertension, dyslipidemia, diabetes mellitus, congenital heart disease, gastrointestinal pathology, dermatological pathology, bone pathology, hypothyroidism, hearing problems, otolaryngology pathology, ophthalmological pathology, psychiatric pathology, epilepsy, and Alzheimer’s disease. Treatment data, with a special focus on the treatment of hypothyroidism, were also collected.

### Biochemical and hematological data

Analyzed biochemical and hematological parameters were selected according to a defined laboratory blood profile as recommended in the guidelines for management of patients with DS^17,20^.

Blood collection and processing were performed in accordance with the Standard Operating Procedures for Serum and Plasma Collection from the Early Detection Research Network (EDRN) Consensus Statement and Standard Operating Procedure Integration Working Group^21^. Blood samples were collected by venipuncture after an overnight fast.

Whole blood samples were collected in VACUTAINER tubes and fractionated by centrifugation at 1,300 *g* for 15 min at room temperature to obtain serum. Serum was aliquoted into 1.5 mL tubes, and the following parameters were measured according to standard commercially available assays adapted to an Architect C4000 (Abbott Diagnostics, USA) using automated procedures: thyroid stimulating hormone (TSH), free thyroxine (FT4), sodium (Na+), potassium (K+), glucose, urea, creatinine, total bilirubin, triglycerides (TG), total cholesterol, aspartate aminotransferase (AST), alanine aminotransferase (ALT), alkaline phosphatase (AF), gamma-glutamyl transferase (GGT), total proteins, vitamin B12, and folate. The estimated glomerular filtration rate (eGFR) was calculated according to the MDRD-4 (Modification of Diet in Renal Disease) and CKD-EPI (Chronic Kidney Disease Epidemiology Collaboration) formulas.

Whole blood samples in EDTA-K_3_ were also obtained for determining blood cell count and indices. The tubes were immediately inverted 10 times to mix the anticoagulant additive with blood. The blood was processed within 2 h of extraction. Using the impedance channel of the automated hematology analyzer Sysmex XE-2100 (Roche Diagnostics, Kobe, Japan), the following parameters were determined: red blood cell count (RBC), white blood cell count (WBC), platelet count, hemoglobin, hematocrit, mean volume (MCV), mean corpuscular hemoglobin concentration (MCHC), mean corpuscular hemoglobin (MCH), red blood cell distribution width (RDW) and mean platelet volume (MPV). The erythrocyte sedimentation rate (ESR) was calculated with a VES cube 200 Sysmex Analyzer (Roche Diagnostics, Kobe, Japan).

Values were compared to normal reference ranges used in our laboratory established in a healthy population from our geographical area according to standardized guides^22^.

### Statistical analysis

Descriptive statistics were used to summarize the characteristics of the study population. Data are presented as medians [25th percentile (P25)–75th percentile (P75)] for continuous variables and as frequencies (percentages) for categorical variables. Data normality was analyzed using the Kolmogorov–Smirnov test. Continuous variables were compared between groups using the Wilcoxon rank-sum test. ANCOVA models, adjusted for age and sex, were used to compare continuous variables across the study groups. Variables were log-transformed to achieve a normal distribution. For clarity, the original values are shown. Categorical variables were compared between groups using Fisher's exact test. Spearman’s rho coefficient was used to assess the correlation between continuous variables. The statistical software package R (https://www.r-project.org) was used for statistical analyses. A P-value < 0.05 was considered statistically significant.

### Ethical aspects

The study was approved by the Sant Pau Ethics Committee following the standards for medical research in humans recommended by the Declaration of Helsinki and in accordance with Spanish legislation for research in people with intellectual disabilities. All participants or their legally authorized representatives gave written informed consent before enrolment in accordance with the guidelines of the local ethics committee.

## Results

### Study cohort characteristics

We enrolled a total of 248 individuals with DS, 132 males (53.2%) and 116 females (46.8%), with a median age of 43.0 (33.0–50.8) years, and 84 control subjects, 21 males (25.0%) and 63 females (75.0%), with a median age of 55.0 (47.3–59.8) years. The clinical features of the DS and control populations are listed in Table 1. The frequency of the following clinical conditions was significantly higher in the DS group than in the control group: history of congenital heart disease, gastrointestinal pathology, dermatological pathology, bone pathology, hypothyroidism, hearing problems, otolaryngology pathology, ophthalmological pathology, epilepsy, and AD. No differences were observed in the frequency of diabetes mellitus or psychiatric pathology for either group. DS individuals presented with a lower frequency of arterial hypertension and dyslipidemia compared to the control group. See Table 1 for further details on the cohort characteristics.

**Table 1:** Characteristics of the Study Population.

	Control		Down syndrome		p-value
	n	Median (P25–P75)/n (%)	n	Median (P25-P754)/n (%)	
Age (years)	84	55.0 (47.3–59.8)	248	43.0 (33.0–50.8)	< 0.001
Male/female	84	21 (25.0)/63 (75.0)	248	132 (53.2)/116 (46.8)	< 0.001
Arterial hypertension	84	16 (19.0)	248	3 (1.2)	< 0.001
Dyslipidemia	84	24 (28.6)	248	37 (14.9)	0.009
Diabetes mellitus	84	6 (7.1)	248	6 (2.4)	0.082
Congenital heart disease	84	0 (0.0)	247	47 (19.0)	< 0.001
Gastrointestinal pathology	84	1 (1.2)	247	40 (16.2)	< 0.001
Dermatological pathology	84	2 (2.4)	247	83 (33.6)	< 0.001
Bone pathology	84	3 (3.6)	247	36 (14.6)	0.006
Hypothyroidism	84	1 (1.2)	247	119 (48.2)	< 0.001
Treatment for hypothyroidism	–		246	103 (41.9)	
Hearing problems	84	0 (0.0)	245	40 (16.3)	< 0.001
Otolaryngology pathology	84	1 (1.2)	247	37 (15.0)	< 0.001
Ophtalmological pathology	84	1 (1.2)	246	136 (55.3)	< 0.001
Psychiatric pathology	84	18 (21.4)	246	45 (18.3)	0.524
Epilepsy	84	1 (1.2)	247	38 (15.4)	< 0.001
Alzheimer’s disease	84	0 (0.0)	247	50 (20.2)	< 0.001

Data are presented as frequencies (percentages) for categorical variables. Continuous variables are presented as median (interquartile range). Differences between groups were analyzed using Wilcoxon rank-sum test or Fisher's exact test.

### Biochemical and hematological parameters in patients with Down syndrome

We performed a detailed biochemical and hematological analysis of the DS cohort and compared the profiles obtained with our control population. The reference values of the studied parameters, the number and percentage of patients out of range, and the median (P25–P75) of the whole study population are shown in Table 2. Seventy-three percent of the studied hematological parameters and 53% of the studied biochemical parameters were significantly different between the DS individuals and the control population. The DS individuals presented with higher TSH, urea, creatinine, AST, hemoglobin, hematocrit, MCV, ESR, MCH and RDW values and lower TG, total cholesterol, folate, eGFR, MPV and WBC values. These differences remained significant, or close to signification, after adjusting for confounding factors such as age and sex. Statistical differences for RBC and MCHC were observed after adjustment. An additional analysis to evaluate the impact of hypothyroidism treatment on TSH was performed.

**Table 2:** Biochemical and hematological parameters in the control group and the cohort of patients with Down Syndrome.

Variable	Reference values		Control			Down syndrome			p-value (categorical)	p-value (continuous)	p-value (continuous, adjusted)
			n	n OOR (%)	Median (P25–P75)	n	n OOR (%)	Median P25–P75			
**Biochemical parameters**											
TSH (mUI/L)	(0.3–5.0)		84	1 (1.2)	1.2 (0.97–1.73)	247	46 (18.6)	2.8 (1.67–4.15)	< 0.001	< 0.001	< 0.001
Na+ (mmol/L)	(136–145)		84	1 (1.2)	140.0 (139.0–141.0)	248	4 (1.6)	140.0 (139.0–141.0)	1.000	0.696	0.889
K+ (mmol/L)	(3.5–5.1)		83	4 (4.8)	4.3 (4.0–4.5)	245	1 (0.4)	4.3 (4.1–4.5)	0.016	0.236	0.472
Glucose (mmol/L)	(3.0–6.1)		84	3 (3.6)	5.0 (4.7–5.4)	248	17 (6.9)	5.0 (4.7–5.3)	0.426	0.559	0.649
Urea (mmol/L)	≤ 60 years (2.1–7.1) > 60 years (2.9–8.2)		84	11 (13.1)	5.5 (4.6–6.3)	248	62 (25.0)	6.1 (5.4–7.2)	0.023	< 0.001	< 0.001
Creatinine (µmol/L)	Females (< 80) Males (< 106)		84	2 (2.4)	64.0 (59.0–72.0)	248	24 (9.7)	74.0 (66.0–84.0)	0.033	< 0.001	< 0.001
eGFR (ml/min/1.73)	(< 60)		84	0 (0.0)	97.4 (91.6–102.2)	248	9 (3.6)	90.0 (84.2–90.0)	0.119	< 0.001	< 0.001
Total bilirrubin (µmol/L)	(< 17)		84	8 (9.5)	9.0 (7.0–11.2)	234	30 (12.8)	10.0 (7.0–13.0)	0.557	0.141	0.778
TG (mmol/L)	(< 1.65)		84	14 (16.7)	0.99 (0.71–1.52)	248	19 (7.7)	0.87 (0.71–1.05)	0.033	0.020	0.004
Total cholesterol (mmol/L)	(< 6.2)		84	20 (23.8)	5.4 (4.8–6.2)	248	15 (6.0)	4.9 (4.3–5.4)	< 0.001	< 0.001	0.027
AST (U/L)	Females (< 31) Males (< 37)		84	10 (11.9)	19.0 (17.0–23.8)	245	12 (4.9)	21.0 (18.0–25.0)	0.040	0.008	0.089
ALT (U/L)	Females (< 31) Males (< 41)		84	10 (11.9)	19.0 (15.0–25.5)	248	25 (10.1)	20.0 (15.0–27.0)	0.682	0.648	0.783
AF (U/L)	Females (35–110) Males (40–130)		78	6 (7.7)	79.0 (63.5–95.5)	247	14 (5.7)	76.0 (64.0–87.0)	0.589	0.609	0.281
GGT (U/L)	Females (< 43) Males (< 54)		84	12 (14.3)	19.0 (14.0–31.5)	248	20 (8.1)	18.0 (13.0–26.0)	0.132	0.092	0.149
Total proteins (g/L)	(64–83)		84	2 (2.4)	69.6 (67.8–71.7)	233	24 (10.3)	68.5 (65.7–71.2)	0.021	0.016	0.021
B12 (pmol/L)	(150–650)		84	7 (8.3)	297.0 (235.0–401.3)	242	10 (4.1)	287.5 (221.8–350.3)	0.156	0.091	0.007
Folate (nmol/L)	(7–45)		84	6 (7.1)	14.7 (11.7–21.6)	243	16 (6.6)	12.3 (9.4–18.3)	0.805	0.009	0.488
**Hematological parameters**											
Hemoglobin (g/L)	Females (120–150) Males (130–170)		82	7 (8.5)	136.0 (128.0–142.0)	248	32 (12.9)	144.0 (136.0–154.0)	0.330	< 0.001	0.005
Hematocrit (L/L)	Females (0.35–0.45) Males (0.4–0.5)		82	6 (7.3)	0.40 (0.38–0.42)	248	23 (9.3)	0.43 (0.41–0.46)	0.660	< 0.001	< 0.001
RBC (× 10^12^/L)	Females (3.9–5) Males (4.5–5.7)		82	9 (11.0)	4.5 (4.3–4.7)	248	58 (23.4)	4.6 (4.2–4.9)	0.017	0.915	0.002
MCV (fL)	(80–98)		82	1 (1.2)	88.4 (86.3–90.5)	248	60 (24.2)	95.4 (92.2–97.8)	< 0.001	< 0.001	< 0.001
ESR (mm/h)	(1–10)		59	32 (54.2)	14.0 (5.0–25.0)	179	137 (76.5)	24.0 (11.0–39.0)	0.002	< 0.001	< 0.001
MCHC (g/L)	(320–360)		82	2 (2.4)	337.0 (330.0–345.0)	248	17 (6.9)	337.0 (330.3–343.0)	0.176	0.940	0.049
MCH (pg)	(27–32)		82	8 (9.8)	29.9 (28.8–30.8)	248	137 (55.2)	32.2 (31.0–33.0)	< 0.001	< 0.001	< 0.001
RDW (%)	(12–15)		82	10 (12.2)	13.0 (12.3–13.7)	248	30 (12.1)	13.7 (13.2–14.4)	1.000	< 0.001	< 0.001
Platelet count (× 10^9^/L)	(140–350)		81	4 (4.9)	251.0 (210.0–275.0)	248	9 (3.6)	235.0 (202.0–274.0)	0.531	0.109	0.054
MPV (fL)	(7.0–10.5)		82	7 (8.5)	8.4 (7.7–9.2)	248	18 (7.3)	7.8 (7.4–8.3)	0.810	< 0.001	0.002
WBC (× 10^9^/L)	(3.8–11.0)		82	3 (3.7)	6.2 (5.3–7.7)	248	30 (12.1)	5.2 (4.4–6.3)	0.032	< 0.001	< 0.001

Differences between groups were analyzed using Wilcoxon Rank-sum test, ANCOVA models adjusted for age and sex, or the Fisher’s exact test.

*OOR* out of range, *NA* not applicable.

No differences were observed for TSH between both studied groups (treated DS individuals = 3.02 (1.25–4.27) vs. untreated DS individuals = 3.20 (1.84–3.98), P-value = 0.194). For categorical variables, the percentage of DS individuals out of range for some parameters was also statistically significant compared to the control population. Parameters with a higher percentage of values out of range in the DS group were TSH, urea, creatinine, total proteins, RBC, MCV, ESR, MCH, and WBC, whereas those with a lower percentage of values out of range were K+ , TG, total cholesterol, and AST.

The differences in the biochemical and hematological parameters and the number and percentage of patients out of range between DS individuals and the control population according to sex are displayed in Supplemental Tables 1 and 2. For the female DS cohort, parameters with significantly higher values were TSH, urea, creatinine, AST, hemoglobin, hematocrit, MCV, ESR, MCH, and RDW, whereas those with significantly lower values were TG, total cholesterol, GGT, eGFR, RBC, MPV, and WBC. For categorical variables, parameters with significantly higher percentages of values out of range were TSH, creatinine, total proteins, MCV, ESR, MCH and WBC, whereas those with a significantly lower percentage of values out of range were total cholesterol and B12 (Supplemental Table 1). For the male DS cohort, parameters with significantly higher values were TSH, hemoglobin, hematocrit, MCV, ESR, MCH, and RDW, whereas those with significantly lower values were TG, total cholesterol, eGFR, MPV, and WBC. Regarding categorical variables, parameters with significantly higher percentages of values out of range were TSH, ESR, and MCH, whereas those with significantly lower percentages of values out of range were K+ , TG, total cholesterol, and GGT (Supplemental Table 2).

The differences in the biochemical and hematological parameters between males and females as well as the frequency and percentage of patients out of range in the control and DS groups are displayed in Supplemental Table 3 and Table 3, respectively. For the control group, parameters with significantly higher values in the male subgroup were K+ , creatinine, TG, ALT, hemoglobin, hematocrit, RBC, and MCHC, whereas those with significantly lower values were AF, eGFR, and ESR. Among the categorical variables, K+ had a significantly higher percentage of values out of range in the male subgroup, and ESR had a significantly lower percentage of values out of range (Supplemental Table 3). For the DS cohort, parameters with significantly higher values in the male subgroup were creatinine, total bilirubin, TG, ALT, GGT, hemoglobin, hematocrit, RBC, MCHC and WBC, whereas those with significantly lower values were folate, MCV, ESR, RDW, platelet count, and MPV. Regarding categorical variables, parameters with significantly higher percentages of values out of range in the male subgroup were total bilirubin, B12, RBC and MPV, whereas those with significantly lower percentages of values out of range were MCV, ESR and MCHC (Table 3).

**Table 3:** Differences between sex in the Down syndrome group.

Variable	Female			Male			p-value (categorical)	p-value (continuous)
	n	n OOR (%)	Median (P25–P75)	n	n OOR (%)	Median (P25–P75)		
**Biochemical parameters**								
TSH (mUI/L)	115	24 (20.9)	2.79 (1.67–4.15)	132	22 (16.7)	2.85 (1.68–3.81)	0.417	0.877
Na+ (mmol/L)	116	1 (0.9)	140.0 (139.0–141.0)	132	3 (2.3)	140.0 (139.0–141.0)	0.625	0.277
K+ (mmol/L)	113	1 (0.9)	4.3 (4.2–4.5)	132	0 (0.0)	4.3 (4.1–4.6)	0.461	0.871
Glucose (mmol/L)	116	4 (3.4)	4.9 (4.6–5.2)	132	13 (9.8)	5.1 (4.8–5.4)	0.075	0.023
Urea (mmol/L)	116	29 (25.0)	6.2 (5.2–7.2)	132	33 (25.0)	6.0 (5.4–7.2)	1.000	0.861
Creatinine (µmol/L)	116	13 (11.2)	68.0 (62.0–75.0)	132	11 (8.3)	82.0 (73.0–94.0)	0.521	< 0.001
eGFR (ml/min/1.73)	116	4 (3.4)	90.0 (83.1–90.0)	132	5 (3.8)	90.0 (87.0–90.0)	1.000	0.179
Total bilirrubin (µmol/L)	111	9 (8.1)	9.0 (6.0–11.0)	123	21 (17.1)	10.0 (8.0–14.0)	0.050	< 0.001
TG (mmol/L)	116	6 (5.2)	0.82 (0.70–0.95)	132	13 (9.8)	0.92 (0.72–1.21)	0.232	0.004
Total cholesterol (mmol/L)	116	9 (7.8)	4.9 (4.5–5.4)	132	6 (4.5)	4.9 (4.2–5.3)	0.302	0.315
AST (U/L)	113	8 (7.1)	22.0 (18.0–26.0)	132	4 (3.0)	21.0 (19.0–25.0)	0.234	0.754
ALT (U/L)	116	13 (11.2)	18.0 (14.0–25.0)	132	12 (9.1)	23.0 (16.0–29.0)	0.674	0.002
AF (U/L)	115	10 (8.7)	76.0 (65.0–88.0)	132	4 (3.0)	76.5 (64.0–87.0)	0.095	0.620
GGT (U/L)	116	9 (7.8)	16.0 (12.0–22.0)	132	11 (8.3)	19.0 (15.0–27.8)	1.000	0.001
Total proteins (g/L)	110	16 (14.5)	68.5 (65.3–71.4)	123	8 (6.5)	68.5 (66.0–70.9)	0.053	0.856
B12 (pmol/L)	112	1 (0.9)	292.0 (227.3–366.5)	130	9 (6.9)	286.5 (208.0–339.5)	0.022	0.278
Folate (nmol/L)	113	7 (6.2)	13.9 (9.9–20.6)	130	9 (6.9)	11.6 (8.8–16.8)	1.000	0.003
**Hematological parameters**								
Hemoglobin (g/L)	116	18 (15.5)	139.0 (132.0–145.0)	132	14 (10.6)	152.0 (143.0–160.0)	0.262	< 0.001
Hematocrit (L/L)	116	10 (8.6)	0.42 (0.39–0.43)	132	13 (9.8)	0.45 (0.42–0.47)	0.828	< 0.001
RBC (× 10^12^/L)	116	17 (14.7)	4.3 (4.1–4.6)	132	41 (31.1)	4.7 (4.4–5.0)	0.003	< 0.001
MCV (fL)	116	37 (31.9)	96.2 (93.0–98.6)	132	23 (17.4)	94.6 (91.8–97.0)	0.011	0.004
ESR (mm/h)	81	75 (92.6)	32.0 (21.5–47.5)	98	62 (63.3)	14.5 (7.0–30.5)	< 0.001	< 0.001
MCHC (g/L)	116	12 (10.3)	334.0 (329.3–341.0)	132	5 (3.8)	338.5 (332.0–346.0)	0.047	0.003
MCH (pg)	116	71 (61.2)	32.4 (30.9–33.3)	132	66 (50.0)	32.1 (31.0–32.7)	0.096	0.189
RDW (%)	116	18 (15.5)	13.9 (13.4–14.6)	132	12 (9.1)	13.6 (13.1–14.3)	0.171	0.016
Platelet count (× 10^9^/L)	116	3 (2.6)	240.0 (216.0–289.0)	132	6 (4.5)	232.0 (196.0–260.8)	0.508	0.024
MPV (fL)	116	3 (2.6)	7.9 (7.5–8.4)	132	15 (11.4)	7.8 (7.3–8.3)	0.012	0.040
WBC (× 10^9^/L)	116	19 (16.4)	5.1 (4.2–6.0)	132	11 (8.3)	5.5 (4.5–6.7)	0.078	0.028

Differences between groups were analyzed using Wilcoxon Rank-sum test or the Fisher’s exact test.

*OOR* out of range, *NA* not applicable.

The correlation between the biochemical and hematological data with age was also explored in both study groups. As shown in Table 4, for the control population, urea, creatinine, total cholesterol and AST showed a significant positive correlation with age, while eGFR showed a significant negative correlation. For the DS population, Na+ , urea, creatinine, TG, total cholesterol, AST, AF, MCV, ESR, MCH, and RDW showed a significant positive correlation with age, while eGFR, ALT, B12, hemoglobin, hematocrit, RBC, MCHC, and platelet count showed a significant negative correlation.

**Table 4:** Correlations between biochemical and hematological parameters and age.

	Control			Down syndrome		
	n	Spearman’s rho	p-value	n	Spearman’s rho	p-value
**Biochemical parameters**						
TSH (mUI/L)	84	0.186	0.090	247	− 0.018	0.777
Na+ (mmol/L)	84	0.142	0.197	248	0.198	0.002
K+ (mmol/L)	83	0.150	0.175	245	0.120	0.060
Glucose (mmol/L)	84	0.163	0.139	248	0.087	0.170
Urea (mmol/L)	84	0.283	0.009	248	0.253	< 0.001
Creatinine (µmol/L)	84	0.319	0.003	248	0.176	0.005
eGFR (ml/min/1.73)	84	− 0.727	< 0.001	248	− 0.498	< 0.001
Total bilirrubin (µmol/L)	84	− 0.006	0.953	234	− 0.017	0.801
TG (mmol/L)	84	0.023	0.838	248	0.150	0.018
Total cholesterol (mmol/L)	84	0.265	0.015	248	0.269	< 0.001
AST (U/L)	84	0.267	0.014	245	0.142	0.026
ALT (U/L)	84	0.158	0.152	248	− 0.153	0.016
AF (U/L)	78	− 0.070	0.541	247	0.147	0.021
GGT (U/L)	84	0.206	0.060	248	0.027	0.671
Total Proteins (g/L)	84	− 0.026	0.811	233	− 0.066	0.315
B12 (pmol/L)	84	0.150	0.173	242	− 0.202	0.002
Folate (nmol/L)	84	0.187	0.088	243	− 0.102	0.113
**Hematological parameters**						
Hemoglobin (g/L)	82	0.195	0.079	248	− 0.162	0.011
Hematocrit (L/L)	82	0.161	0.148	248	− 0.129	0.042
RBC (× 10^12^/L)	82	0.148	0.185	248	− 0.218	0.001
MCV (fL)	82	− 0.022	0.843	248	0.291	< 0.001
ESR (mm/h)	59	− 0.095	0.474	179	0.305	< 0.001
MCHC (g/L)	82	0.092	0.413	248	− 0.160	0.011
MCH (pg)	82	0.047	0.675	248	0.153	0.016
RDW (%)	82	− 0.003	0.979	248	0.172	0.007
Platelet count (× 10^9^/L)	81	− 0.053	0.640	248	− 0.235	< 0.001
MPV (fL)	82	− 0.090	0.424	248	0.124	0.051
WBC (× 10^9^/L)	82	− 0.089	0.428	248	− 0.031	0.629

*NA* not applicable.

## Discussion

The present study evaluated several biochemical and hematological parameters in a large sample of adults with DS. Several studies exploring laboratory tests in DS patients exist, but their focus is limited to specific areas of metabolism^11–16^. DS is among the most complex genetic conditions compatible with life, characterized by accelerated aging and affecting gene expression beyond chromosome 21^23^. The sheer number of affected genes and epigenetic changes suggests that numerous pathways of human metabolism are altered and subsequently might be reflected in laboratory test parameters. Here, we performed a comprehensive approach by analyzing parameters related to different physiological mechanisms. We found significant differences with respect to non-trisomic controls in both biochemical and hematological parameters, even after adjusting for potential confounding factors. Furthermore, we found age- and sex-related differences in several of the parameters. The fact that women with DS experience menopause earlier than healthy women^24^ may explain some of these sex-related differences.

Clinically and as previously described^4,8,9,25^, our DS cohort presented with a higher incidence of congenital heart disease, gastrointestinal pathology, dermatological pathology, bone pathology, hypothyroidism, otolaryngology pathology, ophthalmological pathology, epilepsy and AD than the control population. Arterial hypertension and dyslipidemia were less prevalent, whereas no difference was observed regarding the diabetes mellitus incidence, as discussed below.

With respect to laboratory studies, the hematological profile was largely altered in DS individuals when compared to the control population. Of note, significant differences were found for almost all the hematological parameters when comparing males and females, suggesting the need to consider sex when evaluating the hematological profile in a DS individual. It is well known that trisomy 21 impacts hematopoietic cell biology through multiple and complex pathways. In adults, the metabolic and redox derangements observed in the RBCs from individuals with DS have been previously linked to alterations in cell survival and size, in particular macrocytosis^26^. Different studies have also proposed that the additional copy of chromosome 21 has a profound impact on fetal hematopoiesis, which ultimately impacts the function and number of hematopoietic lineages^27–31^. Additionally, between 4 and 10% of newborn infants with DS develop transient myeloproliferative disorder^32–34^. Although the disease usually resolves without treatment in the first few months of life, it is estimated that 20–30% of individuals with transient myeloproliferative disorder will go on to develop subsequent leukemia^35,36^. Finally, the fact that folate concentrations are significantly lower in DS individuals matches the observed hematological alterations. Taken together, these impaired hematological parameters suggest the existence of abnormalities in hematopoiesis and provide information on how an extra copy of chromosome 21 may lead to phenotypic consequences.

Concerning the biochemical profile, our results support the findings from previous independent studies. We showed that 18.6% of our DS individuals presented with values out of range for TSH level. Of those, 103 out of 119 were treated for hypothyroidism. Impaired TSH and FT4 levels have been largely described in DS populations^37^. Moreover, subclinical hypothyroidism in children with DS is an abundantly common occurrence, with a prevalence of approximately 30%^38^, and has been attributed to the dysregulation of the hypothalamic-pituitary-thyroid axis^37^. Regarding urea metabolism, 25% of our DS individuals presented with a high urea concentration, which may be due to impaired renal function, among other causes. Indeed, and as previously reported^39^, almost 10% of our DS individuals also presented with impaired creatinine values. Serum creatinine is the most reliable parameter for detecting kidney damage due to its high diagnostic specificity. From its concentration and based on formulas in which age, sex and weight are taken into account, it is possible to estimate the glomerular filtration rate (eGFR). Our DS cohort also presented with a lower eGFR, which is in agreement with a previous study exploring renal disease in DS individuals^40^. Despite the significantly altered parameters related to renal function, our DS individuals presented with a very low frequency of arterial hypertension.

Concerning the lipid profile, we found significantly lower total cholesterol and TG concentrations in DS individuals compared to the control population. It would have been interesting to study the fractioned forms of cholesterol together with their apolipoprotein concentrations; however, because the current study was not designed to answer questions regarding lipid metabolism, low-density lipoprotein cholesterol (LDLc) and high-density lipoprotein cholesterol (HDLc) were not measured. Several works measuring circulating total cholesterol, LDLc, HDLc and TG concentrations in the DS population exist. However, they report contradictory results and prevent firm conclusions from being drawn. Some studies have reported an unfavorable^41–45^ or favorable lipid profile^46^. However, most of the studies reported no change in serum TC, LDLc or HDLc in individuals with DS compared to a control group or to population norms^41,45,47–51^. In our study, these lower total cholesterol and TG concentrations may have translated into a significantly lower prevalence of hyperlipidemia in DS individuals. It has been described that DS individuals may be protected against atherosclerosis^47,52–54^, leading to a low incidence of cardiovascular events^53^. However, a work carried out with 4,081 individuals with DS found that they were at high risk of cerebrovascular events, but a lower risk of coronary events in males^55^. Therefore, risk of major cerebrovascular events in people with DS should not be ruled out. Concerning diabetes mellitus, a similar incidence of type 2 diabetes mellitus^50^ and a higher incidence of type 1 diabetes mellitus has been described for individuals with DS^56^. We found no difference in type 1 diabetes mellitus frequency among our DS and control populations as previously described in a different study^16^. In regard to arterial hypertension prevalence, our results are in line with numerous studies that have described a lower incidence of this condition in DS individuals^50,51,57,58^. Despite these observations, cholesterol fractioned forms and glycated hemoglobin (HbA1c) concentrations were not measured, making it difficult to draw conclusions regarding dyslipidemia and diabetes mellitus in our cohort. Yet, an increased degree of hypolipidemia should not be ruled out. Overall, future studies elucidating the mechanisms behind the low cholesterol and TG concentrations and lower prevalence of arterial hypertension observed in our DS cohort should be performed.

It is important to emphasize that our main goal was to help determining if the observed biochemical and hematological alterations have direct clinical implications for DS individuals. While the altered biochemical and hematological profiles may be developmental features (i.e., a consequence of the specific genetic characteristics of individuals with DS) or the result of accelerated aging, it should be recalled that they may also be reflecting comorbidities or the use of medication. From a clinical standpoint, to elucidate if the observed differences are consequence of concomitant conditions or features of the syndrome itself could be of help in the management of DS individuals. Unfortunately, due to the design of our study, these questions remain unanswered. Future studies focusing on specific areas of metabolism of DS individuals with different comorbidities could shed some light on this matter.

Our study has several strengths. We collected relevant clinical, biochemical and hematological data in a large DS cohort and performed a systematic analysis. The fact that our controls were chosen from a healthy background broadens the actual differences and strengthens the present results. Ultimately, according to the wide inclusion criteria and the broad range of represented ages, we believe that the results from our study may help clinicians when interpreting laboratory analyses in DS individuals. Some limitations should also be taken into account. The control and DS populations were not strictly age and sex matched and the control group had a reduced number of males when compared to females. Nonetheless, both populations were within the same age range and additional analysis including adjustment for age and sex were performed. Furthermore, despite our large cohort of DS individuals, the number was still not sufficient to perform statistical analysis stratification according to the observed clinical conditions. Moreover and as stated previously, some of the observed biochemical and/or hematological alterations may have been a consequence of the use of drugs for the treatment of other comorbidities. Finally, our defined clinical, biochemical and hematological profiles were somehow general and unable to cover all the possible comorbidities present in DS individuals.

In conclusion, adults with DS show a specific profile of biochemical and hematological parameters. A good understanding of the differences in our cohort with those in the general population might aid in the clinical follow-up of adults with DS, especially considering that the life span of DS individuals can now reach 60 years of age in developed countries.

## Supplementary information

Supplementary Information.

## Abbreviations

AD: Alzheimer’s disease
AF: Alkaline phosphatase
ALT: Alanine aminotransferase
AST: Aspartate aminotransferase
B12: Vitamin B12
CKD-EPI: Chronic kidney disease epidemiology collaboration
DS: Down syndrome
ESR: Erythrocyte sedimentation rate
FT4: Free thyroxine
eGFR: Estimated glomerular filtration rate
GGT: Gamma-glutamyl transferase
HbA1c: Glycated hemoglobin
HDLc: High-density lipoprotein cholesterol
LDLc: Low-density lipoprotein cholesterol
MCH: Mean corpuscular hemoglobin
MCHC: Mean corpuscular hemoglobin concentration
MCV: Mean corpuscular volume
MDRD-4: Modification of diet in renal disease
MPV: Mean platelet volume
K+: Potassium
RDW: Red blood cell distribution width
Na+: Sodium
TG: Triglycerides
TSH: Thyroid stimulating hormone
