# Supplementary material for: Evaluation of biochemical and hematological parameters in adults with Down syndrome
Source: Sci Rep. 2020 Aug 13;10:13755. doi: 10.1038/s41598-020-70719-2 (PMC7426851; doi:10.1038/s41598-020-70719-2)
Supplement: Supplementary file 1 — Supplementary Information. [file 41598_2020_70719_MOESM1_ESM.docx]

| Supplemental Table 1. Biochemical and hematological parameters in the control group and the cohort of patients with Down Syndrome (Females). | | | | | | | | |
| --- | --- | --- | --- | --- | --- | --- | --- | --- |
|  | **CONTROL** | | | **DOWN SYNDROME** | | |  |  |
| Variable | **n** | **n OOR (%)** | **Median (P25-P75)** | **n** | **n OOR (%)** | **Median (P25-P75)** | **p-value**  **(categorical)** | **p-value**  **(continuous)** |
| Biochemical Parameters |  |  |  |  |  |  |  |  |
| TSH (mUI/L) | 63 | 1 (1.6) | 1.31 (0.97-1.73) | 115 | 24 (20.9) | 2.79 (1.67-4.15) | <0.001 | <0.001 |
| Na+ (mmol/L) | 63 | 1 (1.6) | 140.0 (139.0-141.0) | 116 | 1 (0.9) | 140.0 (139.0-141.0) | 1000 | 0.610 |
| K+ (mmol/L) | 62 | 1 (1.6) | 4.3 (3.9-4.5) | 113 | 1 (0.9) | 4.3 (4.2-4.5) | 1000 | 0.062 |
| Glucose (mmol/L) | 63 | 1 (1.6) | 5.0 (4.5-5.3) | 116 | 4 (3.4) | 4.9 (4.6-5.2) | 0.658 | 0.746 |
| Urea (mmol/L) | 63 | 9 (14.3) | 5.2 (4.3-6.5) | 116 | 29 (25.0) | 6.2 (5.2-7.2) | 0.125 | 0.001 |
| Creatinine (µmol/L) | 63 | 1 (1.6) | 62.0 (57.0-67.0) | 116 | 13 (11.2) | 68.0 (62.0-75.0) | 0.021 | <0.001 |
| eGFR (ml/min/1.73) | 63 | 0 (0.0) | 98.3 (93.2-103.6) | 116 | 4 (3.4) | 90.0 (83.1-90.0) | 0.299 | <0.001 |
| Total Bilirrubin (µmol/L) | 63 | 6 (9.5) | 8.5 (6.8-11.0) | 111 | 9 (8.1) | 9.0 (6.0-11.0) | 0.783 | 0.934 |
| TG (mmol/L) | 63 | 8 (12.7) | 0.94 (0.70-1.37) | 116 | 6 (5.2) | 0.82 (0.70-0.95) | 0.086 | 0.040 |
| Total Cholesterol (mmol/L) | 63 | 13 (20.6) | 5.3 (4.7-6.1) | 116 | 9 (7.8) | 4.9 (4.5-5.4) | 0.017 | 0.005 |
| AST (U/L) | 63 | 8 (12.7) | 19.0 (16.0-23.0) | 113 | 8 (7.1) | 22.0 (18.0-26.0) | 0.275 | 0.013 |
| ALT (U/L) | 63 | 9 (14.3) | 18.0 (14.0-23.0) | 116 | 13 (11.2) | 18.0 (14.0-25.0) | 0.635 | 0.955 |
| AF (U/L) | 60 | 6 (10.0) | 82.5 (65.3-102.5) | 115 | 10 (8.7) | 76.0 (65.0-88.0) | 0.787 | 0.210 |
| GGT (U/L) | 63 | 7 (11.1) | 18.0 (14.0-31.0) | 116 | 9 (7.8) | 16.0 (12.0-22.0) | 0.584 | 0.046 |
| Total Proteins (g/L) | 63 | 1 (1.6) | 69.6 (67.8-71.7) | 110 | 16 (14.5) | 68.5 (65.3-71.4) | 0.006 | 0.076 |
| B12 (pmol/L) | 63 | 6 (9.5) | 298.0 (252.0-419.0) | 112 | 1 (0.9) | 292.0 (227.3-366.5) | 0.009 | 0.105 |
| Folate (nmol/L) | 63 | 4 (6.3) | 15.0 (11.7-24.6) | 113 | 7 (6.2) | 13.9 (9.9-20.6) | 1.000 | 0.242 |
| Hematological Parameters |  |  |  |  |  |  |  |  |
| Hemoglobin (g/L) | 62 | 7 (11.3) | 133.0 (126.8-139.3) | 116 | 18 (15.5) | 139.0 (132.0-145.0) | 0.504 | <0.001 |
| Hematocrit (L/L) | 62 | 4 (6.5) | 0.40 (0.38-0.41) | 116 | 10 (8.6) | 0.42 (0.39-0.43) | 0.773 | <0.001 |
| RBC (x10^12^/L) | 62 | 6 (9.7) | 4.4 (4.3-4.6) | 116 | 17 (14.7) | 4.3 (4.1-4.6) | 0.482 | 0.033 |
| MCV (fL) | 62 | 0 (0.0) | 88.3 (86.7-90.5) | 116 | 37 (31.9) | 96.2 (93.0-98.6) | <0.001 | <0.001 |
| ESR (mm/h) | 46 | 29 (63.0) | 17.0 (7.8-26.3) | 81 | 75 (92.6) | 32.0 (21.5-47.5) | <0.001 | <0.001 |
| MCHC (g/L) | 62 | 2 (3.2) | 335.5 (328.8-343.3) | 116 | 12 (10.3) | 334.0 (329.3-341.0) | 0.143 | 0.713 |
| MCH (pg) | 62 | 6 (9.7) | 29.7 (28.7-30.7) | 116 | 71 (61.2) | 32.4 (30.9-33.3) | <0.001 | <0.001 |
| RDW (%) | 62 | 8 (12.9) | 13.1 (12.4-13.7) | 116 | 18 (15.5) | 13.9 (13.4-14.6) | 0.824 | <0.001 |
| Platelet count (x10^9^/L) | 61 | 4 (6.6) | 253.0 (210.5-278.0) | 116 | 3 (2.6) | 240.0 (216.0-289.0) | 0.235 | 0.341 |
| MPV (fL) | 62 | 4 (6.5) | 8.4 (7.7-9.2) | 116 | 3 (2.6) | 7.9 (7.5-8.4) | 0.240 | 0.004 |
| WBC (x10^9^/L) | 62 | 2 (3.2) | 6.0 (5.1-7.5) | 116 | 19 (16.4) | 5.1 (4.2-6.0) | 0.013 | <0.001 |
| Differences between groups were analyzed using Wilcoxon Rank-sum test or the Fisher’s exact test. OOR: Out of Range; NA: Not Applicable. | | | | | | | | |

| Supplemental Table 2. Biochemical and hematological parameters in the control group and the cohort of patients with Down syndrome (Males) | | | | | | | | |
| --- | --- | --- | --- | --- | --- | --- | --- | --- |
|  | **CONTROL** | | | **DOWN SYNDROME** | | |  |  |
| Variable | **n** | **n OOR (%)** | **Median (P25-P75)** | **n** | **n OOR (%)** | **Median (P25-P75)** | **p-value**  **(categorical)** | **p-value**  **(continuous)** |
| Biochemical Parameters | | | | | | | | |
| TSH (mUI/L) | 21 | 0 (0.0) | 1.04 (0.91-1.61) | 132 | 22 (16.7) | 2.85 (1.68-3.81) | 0.045 | <0.001 |
| Na+ (mmol/L) | 21 | 0 (0.0) | 140.0 (139.0-142.0) | 132 | 3 (2.3) | 140.0 (139.0-141.0) | 1.000 | 0.565 |
| K+ (mmol/L) | 21 | 3 (14.3) | 4.4 (4.1-4.7) | 132 | 0 (0.0) | 4.3 (4.1-4.6) | 0.002 | 0.255 |
| Glucose (mmol/L) | 21 | 2 (9.5) | 5.2 (4.8-5.6) | 132 | 13 (9.8) | 5.1 (4.8-5.4) | 1.000 | 0.483 |
| Urea (mmol/L) | 21 | 2 (9.5) | 5.7 (5.3-6.2) | 132 | 33 (25.0) | 6.0 (5.4-7.2) | 0.163 | 0.225 |
| Creatinine (µmol/L) | 21 | 1 (4.8) | 76.0 (72.0-86.5) | 132 | 11 (8.3) | 82.0 (73.0-94.0) | 1.000 | 0.205 |
| eGFR (ml/min/1.73) | 21 | 0 (0.0) | 93.8 (83.8-99.2) | 132 | 5 (3.8) | 90.0 (87.0-90.0) | 1.000 | <0.001 |
| Total Bilirrubin (µmol/L) | 21 | 2 (9.5) | 10.0 (8.0-11.7) | 123 | 21 (17.1) | 10.0 (8.0-14.0) | 0.528 | 0.321 |
| TG (mmol/L) | 21 | 6 (28.6) | 1.49 (0.86-1.78) | 132 | 13 (9.8) | 0.92 (0.72-1.21) | 0.027 | 0.009 |
| Total Cholesterol (mmol/L) | 21 | 7 (33.3) | 5.8 (5.0-6.4) | 132 | 6 (4.5) | 4.9 (4.2-5.3) | <0.001 | <0.001 |
| AST (U/L) | 21 | 2 (9.5) | 20.6 (17.5-24.7) | 132 | 4 (3.0) | 21.0 (19.0-25.0) | 0.192 | 0.501 |
| ALT (U/L) | 21 | 1 (4.8) | 24.0 (18.0-31.3) | 132 | 12 (9.1) | 23.0 (16.0-29.0) | 1.000 | 0.312 |
| AF (U/L) | 18 | 0 (0.0) | 71.0 (53.3-79.0) | 132 | 4 (3.0) | 76.5 (64.0-87.0) | 1.000 | 0.057 |
| GGT (U/L) | 21 | 5 (23.8) | 27.0 (15.0-46.0) | 132 | 11 (8.3) | 19.0 (15.0-27.8) | 0.047 | 0.093 |
| Total Proteins (g/L) | 21 | 1 (4.8) | 69.4 (67.8-71.9) | 123 | 8 (6.5) | 68.5 (66.0-70.9) | 1.000 | 0.135 |
| B12 (pmol/L) | 21 | 1 (4.8) | 267.0 (218.5-338.0) | 130 | 9 (6.9) | 286.5 (208.0-339.5) | 1.000 | 0.857 |
| Folate (nmol/L) | 21 | 2 (9.5) | 14.6 (11.6-17.7) | 130 | 9 (6.9) | 11.6 (8.8-16.8) | 0.652 | 0.150 |
| Hematological Parameters | | | | | | | | |
| Hemoglobin (g/L) | 20 | 0 (0.0) | 142.5 (139.3-151.5) | 132 | 14 (10.6) | 152.0 (143.0-160.0) | 0.217 | 0.008 |
| Hematocrit (L/L) | 20 | 2 (10.0) | 0.42 (0.40-0.44) | 132 | 13 (9.8) | 0.45 (0.42-0.47) | 1.000 | 0.002 |
| RBC (x10^12^/L) | 20 | 3 (15.0) | 4.7 (4.6-5.1) | 132 | 41 (31.1) | 4.7 (4.4-5.0) | 0.188 | 0.610 |
| MCV (fL) | 20 | 1 (5.0) | 88.7 (85.5-90.9) | 132 | 23 (17.4) | 94.6 (91.8-97.0) | 0.202 | <0.001 |
| ESR (mm/h) | 13 | 3 (23.1) | 6.0 (3.5-13.0) | 98 | 62 (63.3) | 14.5 (7.0-30.5) | 0.007 | 0.017 |
| MCHC (g/L) | 20 | 0 (0.0) | 343.5 (335.0-347.5) | 132 | 5 (3.8) | 338.5 (332.0-346.0) | 1.000 | 0.196 |
| MCH (pg) | 20 | 2 (10.0) | 30.4 (29.1-31.0) | 132 | 66 (50.0) | 32.1 (31.0-32.7) | 0.001 | <0.001 |
| RDW (%) | 20 | 2 (10.0) | 12.7 (12.3-13.3) | 132 | 12 (9.1) | 13.6 (13.1-14.3) | 1.000 | <0.001 |
| Platelet count (x10^9^/L) | 20 | 0 (0.0) | 236.5 (202.5-266.8) | 132 | 6 (4.5) | 232.0 (196.0-260.8) | 1.000 | 0.709 |
| MPV (fL) | 20 | 3 (15.0) | 8.4 (7.3-9.2) | 132 | 15 (11.4) | 7.8 (7.3-8.3) | 0.709 | 0.033 |
| WBC (x10^9^/L) | 20 | 1 (5.0) | 7.1 (5.8-8.3) | 132 | 11 (8.3) | 5.5 (4.5-6.7) | 1.000 | <0.001 |
| Differences between groups were analyzed using Wilcoxon Rank-sum test or the Fisher’s exact test. OOR: Out of Range; NA: Not Applicable. | | | | | | | | |

| Supplemental Table 3. Differences between sex in the control group. | | | | | | | | |
| --- | --- | --- | --- | --- | --- | --- | --- | --- |
|  | **FEMALE** | | | **MALE** | | |  |  |
| Variable | **n** | **n OOR (%)** | **Median (P25-P75)** | **n** | **n OOR (%)** | **Median (P25-P75)** | **p-value**  **(categorical)** | **p-value**  **(continuous)** |
| Biochemical parameters | | | | | | | | |
| TSH (mUI/L) | 63 | 1 (1.6) | 1.31 (0.97-1.73) | 21 | 0 (0.0) | 1.04 (0.91-1.61) | 1.000 | 0.258 |
| Na+ (mmol/L) | 63 | 1 (1.6) | 140.0 (139.0-141.0) | 21 | 0 (0.0) | 140.0 (139.0-142.0) | 1.000 | 0.455 |
| K+ (mmol/L) | 62 | 1 (1.6) | 4.3 (3.9-4.5) | 21 | 3 (14.3) | 4.4 (4.1-4.7) | 0.048 | 0.037 |
| Glucose (mmol/L) | 63 | 1 (1.6) | 5.0 (4.5-5.3) | 21 | 2 (9.5) | 5.2 (4.8-5.6) | 0.153 | 0.063 |
| Urea (mmol/L) | 63 | 9 (14.3) | 5.2 (4.3-6.5) | 21 | 2 (9.5) | 5.7 (5.3-6.2) | 0.723 | 0.126 |
| Creatinine (µmol/L) | 63 | 1 (1.6) | 62.0 (57.0-67.0) | 21 | 1 (4.8) | 76.0 (72.0-86.5) | 0.440 | <0.001 |
| eGFR (ml/min/1.73) | 63 | 0 (0.0) | 98.3 (93.2-103.6) | 21 | 0 (0.0) | 93.8 (83.8-99.2) | NA | 0.034 |
| Total Bilirrubin (µmol/L) | 63 | 6 (9.5) | 8.5 (6.8-11.0) | 21 | 2 (9.5) | 10.0 (8.0-11.7) | 1.000 | 0.213 |
| TG (mmol/L) | 63 | 8 (12.7) | 0.94 (0.70-1.37) | 21 | 6 (28.6) | 1.49 (0.86-1.78) | 0.103 | 0.019 |
| Total Cholesterol (mmol/L) | 63 | 13 (20.6) | 5.3 (4.7-6.1) | 21 | 7 (33.3) | 5.8 (5.0-6.4) | 0.250 | 0.116 |
| AST (U/L) | 63 | 8 (12.7) | 19.0 (16.0-23.0) | 21 | 2 (9.5) | 20.6 (17.5-24.7) | 1.000 | 0.284 |
| ALT (U/L) | 63 | 9 (14.3) | 18.0 (14.0-23.0) | 21 | 1 (4.8) | 24.0 (18.0-31.3) | 0.439 | 0.005 |
| AF (U/L) | 60 | 6 (10.0) | 82.5 (65.3-102.5) | 18 | 0 (0.0) | 71.0 (53.3-79.0) | 0.327 | 0.008 |
| GGT (U/L) | 63 | 7 (11.1) | 18.0 (14.0-31.0) | 21 | 5 (23.8) | 27.0 (15.0-46.0) | 0.164 | 0.090 |
| Total Proteins (g/L) | 63 | 1 (1.6) | 69.6 (67.8-71.7) | 21 | 1 (4.8) | 69.4 (67.8-71.9) | 0.440 | 0.877 |
| B12 (pmol/L) | 63 | 6 (9.5) | 298.0 (252.0-419.0) | 21 | 1 (4.8) | 267.0 (218.5-338.0) | 0.674 | 0.102 |
| Folate (nmol/L) | 63 | 4 (6.3) | 15.0 (11.7-24.6) | 21 | 2 (9.5) | 14.6 (11.6-17.7) | 0.637 | 0.215 |
| Hematological parameters | | | | | | | | |
| Hemoglobin (g/L) | 62 | 7 (11.3) | 133.0 (126.8-139.3) | 20 | 0 (0.0) | 142.5 (139.3-151.5) | 0.186 | <0.001 |
| Hematocrit (L/L) | 62 | 4 (6.5) | 0.40 (0.38-0.41) | 20 | 2 (10.0) | 0.42 (0.40-0.44) | 0.630 | <0.001 |
| RBC (x10^12^/L) | 62 | 6 (9.7) | 4.4 (4.3-4.6) | 20 | 3 (15.0) | 4.7 (4.6-5.1) | 0.681 | <0.001 |
| MCV (fL) | 62 | 0 (0.0) | 88.3 (86.7-90.5) | 20 | 1 (5.0) | 88.7 (85.5-90.9) | 0.244 | 0.931 |
| ESR (mm/h) | 46 | 29 (63.0) | 17.0 (7.8-26.3) | 13 | 3 (23.1) | 6.0 (3.5-13.0) | 0.014 | 0.013 |
| MCHC (g/L) | 62 | 2 (3.2) | 335.5 (328.8-343.3) | 20 | 0 (0.0) | 343.5 (335.0-347.5) | 1.000 | 0.013 |
| MCH (pg) | 62 | 6 (9.7) | 29.7 (28.7-30.7) | 20 | 2 (10.0) | 30.4 (29.1-31.0) | 1.000 | 0.179 |
| RDW (%) | 62 | 8 (12.9) | 13.1 (12.4-13.7) | 20 | 2 (10.0) | 12.7 (12.3-13.3) | 1.000 | 0.133 |
| Platelet count (x10^9^/L) | 61 | 4 (6.6) | 253.0 (210.5-278.0) | 20 | 0 (0.0) | 236.5 (202.5-266.8) | 0.567 | 0.171 |
| MPV (fL) | 62 | 4 (6.5) | 8.4 (7.7-9.2) | 20 | 3 (15.0) | 8.4 (7.3-9.2) | 0.353 | 0.650 |
| WBC (x10^9^/L) | 62 | 2 (3.2) | 6.0 (5.1-7.5) | 20 | 1 (5.0) | 7.1 (5.8-8.3) | 1.000 | 0.057 |
| Differences between groups were analyzed using Wilcoxon Rank-sum test or the Fisher’s exact test. OOR: Out of Range; NA: Not Aplicable. | | | | | | | | |
